# Dr. Walter J. Ransom

**Published:** 1897-11

**Authors:** 


Dr. Walter J. Ransom, of Lockport, who died last August, was a man of more than ordinary ability, and a physician who commanded the respect of his colleagues, neighbors and acquaintances. His demise in the midst of a life of usefulness and in the activity of his years is a sad blow to his family and to an extensive clientèle that he was serving so faithfully and well.

